# Comparison of treatment outcomes of 360° intraoperative laser retinopexy and focal laser retinopexy with pars plans vitrectomy in patients with primary rhegmatogenous retinal detachment

**DOI:** 10.1186/s12886-023-02812-9

**Published:** 2023-02-21

**Authors:** Ying Zheng, Philip Schindler, Vasyl Druchkiv, Jan Schulz, Stephan Martin Spitzer, Christos Skevas

**Affiliations:** 1 Department of Ophthalmology, University Medical Center Hamburg-Eppendorf, Hamburg, Germany; 2 Department of Ophthalmology, Shanghai General Hospital, Shanghai Jiaotong University, 100 Haining Road, 200080 Shanghai, China; 3 Department of Clínica Baviera, Valencia, Spain

**Keywords:** 360° intraoperative laser retinopexy, Primary rhegmatogenous retinal detachment, Focal laser retinopexy

## Abstract

**Background:**

This study was to compare the outcomes of 360° intra-operative laser retinopexy (ILR) and focal laser retinopexy in treating patients with pars plans vitrectomy (PPV) for primary rhegmatogenous retinal detachment (RRD). To identify other potential risk factors for retinal re-detachment after primary PPV.

**Methods:**

This was a retrospective cohort study. Three hundred and forty-four consecutive cases of primary rhegmatogenous retinal detachment treated with PPV were included between July 2013 and July 2018. Clinical characteristics and surgical outcomes were compared between focal laser retinopexy and additional 360° intra-operative laser retinopexy groups. Both univariate and multiple variable analysis were used to identify potential risk factors for retinal re-detachment.

**Results:**

Median follow-up was 6.2 months (Q1, Q3:2.0, 17.2). As estimated with survival analysis, the 360º ILR group had the incidence of 9.74% and focal laser 19.54% at 6 months postoperatively. At 12 months postoperatively the difference was 10.78% vs. 25.21%. The difference in survival rates was significant (p = 0.0021). In multivariate Cox regression, the risk factors for retinal re-detachment were without additional 360° ILR, diabetes and macula off before the primary surgery (relatively OR = 0.456, 95%-CI [0.245–0.848], p < 0.05; OR = 2.301, 95% CI [1.130–4.687], p < 0.05; OR = 2.243, 95% CI [1.212–4.149], p < 0.05).

**Conclusion:**

Additional 360° ILR group had a significantly lower rate of retinal re-detachment when compared with focal laser retinopexy group. Our study also elucidated that diabetes and macular off before the primary surgery might also be the potential risk factors for higher rate of retinal re-detachment outcome.

**Trial registration:**

This was a retrospective cohort study.

## Backgound

Visual-threatening rhegmatogenous retinal detachment (RRD), characterized by the presence of peripheral full-thickness retinal breaks, is the most common form of retinal detachment [1]–[2]. Main surgical treatments for RRD include pars plans vitrectomy (PPV), scleral buckling (SB) and combined techniques [3]–[4]. Small incisional PPV, is growing in popularity as a first-line surgical procedure for RRD [4]. Due to better intraoperative control, PPV has an improved view of retinal periphery, allowing superior identification of retinal breaks [5]–[6].

Anatomical success after primary PPV surgery is very important for RRD treatment, since vision decreases dramatically with subsequent surgeries in most cases. [7]. However, the rate of re-detachment after successful primary surgery varies between 4 to 20% [8–10]. Studies showed that the major causes of retinal re-detachment are missed breaks, opening of old breaks due to persistent or renewed traction or new break formation [11].

Efforts have been made to reduce the risks of peripheral retinal breaks and retinal re-detachments. Additional 360° intraoperative laser retinopexy (ILR) may serve to wall off concealed retinal breaks or any retinal re-detachment anterior to the barrage. It can be easily performed during PPV with the endolaser probe. Several studies have shown a significant decrease of RRD-rate in eyes that underwent PPV for macular disease, retinal detachments following silicone oil removal by using 360° ILR [12–14].

In this study, we evaluate the effect of 360° ILR on the rate of retinal re-detachment following primary PPV for RRD. We also aim to explore other potential risk factors for retinal re-detachment after primary PPV for RRD.

## Methods

Our clinic’s internal electronic patient database was retrospectively searched for patients with RRD treated with 23G PPV between 2013 and 2018. The study followed the tenets of the Declaration of Helsinki and was approved by the Medical Institutional Review Board of Hamburg (PV7315). Informed consent was waivered because of the retrospective design of the study.

Patients were excluded from the study if they had any history of prior retinal detachment, intraocular inflammation like uveitis and endophthalmitis, perforating or contusional trauma, intraocular tumor, proliferative (except in the context of RRD) or exudative retinopathy. Patients with proliferative diabetic retinopathy were also excluded, but non-proliferative diabetic retinopathy did not lead to exclusion.

Patients were included if they underwent a complete ophthalmological examination, including best corrected visual acuity (BCVA) testing using Snellen chart, intraocular pressure measurement, anterior segment examination with slit-lamp biomicroscopy and dilated funduscopy.

Surgeries were performed under local anaesthesia with a retrobulbar block or under general anaesthesia, according to patient preferences. After displacing the conjunctiva, three cannulas were inserted using a bevelled trocar into the inferotemporal, superatemporal, and superanasal quadrants. A 23-gauge infusion cannula was placed at the inferotemporal sclerotomy site. Central and peripheral vitrectomy was performed, and vitreous base shaving was performed in all patients with scleral depression. Retinal breaks were localized and marked with endodiathermy. Fluid/air exchange was then performed. Endolaser treatment was performed under air. In the group where focal laser was performed, ILR was performed around the retinal breaks, holes, and areas of lattice degeneration. In the other group additional 360° ILR was performed as three rows of medium-white burns anterior to the level of the vortex vein, towards, and beyond the equator.

All burns were distanced one burn width apart. The type of tamponade was chosen based on whether the macular was affected by the initial retinal detachment, how many quadrants were affected by initial retinal detachment and the PVR grade.

Follow-up examinations were considered as long as there were entries in the patients’ electronic file meeting the inclusion criteria. A patient’s status on retinal detachment was censored after the last record in the electronic file, leading to different follow-up times at time of data recording. The failure/re-detachment was defined as three months after the initial surgery.

Following parameters have been analyzed for each individual case:

- preoperative parameters.
  - gender (male/female).
  - age at surgery (years).
  - lens status (phakic/pseudophakic/aphakic).
  - macula affected by initial retinal detachment (yes/no).
  - quadrants affected by initial retinal detachment (1 or ≥ 1).
  - degree of proliferative vitreoretinopathy (PVR) at initial retinal detachment (A/B/C/D).
  - axial bulbar length (mm).
  - diabetes (yes/no).
- intraoperative parameters.
  - 360° endolaser cerclage (yes/no).
  - focal endolaser treatment (yes/no).
  - PPV combined with cataract surgery and/or epiretinal membrane peeling.
  - endotamponade used (none/air/SF6 gas/C2F6 gas/C3F8 gas/silicon oil).
- postoperative parameters.
  - retinal re-detachment (1x/2x/3x…).
  - time to retinal re-detachment.
  - degree of proliferative vitreoretinopathy (PVR) at time of re-detachment.
  - occurrence of epimacular gliosis.

The attachment rates presented were after silicone oil removal. The correlation of RRD with the risk factors was analyzed with non-parametric Kaplan-Maier time-to-event estimation. To compute hazard ratios Cox regression was applied. The variables with significant bivariate correlation were analyzed further with multivariate Cox regression.

## Results

### Demographics of patients with retinal re-detachment after primary PPV

The study included 344 consecutive eyes with RRD who underwent primary PPV. There were two cohorts of those consecutive cases: Group 1: the patients without retinal re-detachment after primary PPV (n = 308) and Group 2: the patients with retinal re-detachment after primary PPV (n = 36).

Table 1 summarizes the baseline demographic and clinical characteristics of all the patients including sex, age, diabetes status, with or without 360° ILR, combined operations, endotamponade, surgeons, lens status, macular status PVR grade, RD quadrants, axial length, and duration of follow-ups (shown in Table 1).

**Table 1:** Demographics and clinical characteristics of patients with retinal redetachment * p < 0.1, ** p < 0.05, *** p < 0.01.

	Redetachment			
	**No (N = 308)**	**Yes (N = 36)**	**Total (N = 344)**	**p value**
**Sex**				0.23
Male	202 (91.8%)	18 (8.2%)	220 (100.0%)	
Female	106 (85.5%)	18 (14.5%)	124 (100.0%)	
**Age, yrs**				0.13
Mean (SD)	62.9 (12.9)	60.2 (11.1)	62.6 (12.7)	
Range	18.5–93.9	39.7–81.4	18.5–93.9	
**Diabetes**				0.025**
No	275 (90.8%)	28 (9.2%)	303 (100.0%)	
Yes	33 (80.5%)	8 (19.5%)	41 (100.0%)	
**Laser Cerclage**				0.0021***
No	122 (86.5%)	19 (13.5%)	141 (100.0%)	
Yes	186 (91.6%)	17 (8.4%)	203 (100.0%)	
**Combined operation**				0.41
No	226 (90.0%)	25 (10.0%)	251 (100.0%)	
with cataract-operation	64 (87.7%)	9 (12.3%)	73 (100.0%)	
with peeling	17 (89.5%)	2 (10.5%)	19 (100.0%)	
with IVI	1 (100.0%)	0 (0.0%)	1 (100.0%)	
**Endotamponade**				0.24
SF6 Gas	3 (100.0%)	0 (0.0%)	3 (100.0%)	
C2F6 Gas	98 (85.2%)	17 (14.8%)	115 (100.0%)	
C3F8 Gas	54 (88.5%)	7 (11.5%)	61 (100.0%)	
silicone oil	153 (92.7%)	12 (7.3%)	165 (100.0%)	
**Operator**				0.0078***
S	240 (89.6%)	28 (10.4%)	268 (100.0%)	
W	68 (89.5%)	8 (10.5%)	76 (100.0%)	
**Lens Status**				0.26
aphakia	2 (100.0%)	0 (0.0%)	2 (100.0%)	
phakic	102 (91.1%)	10 (8.9%)	112 (100.0%)	
pseudophakic	204 (88.7%)	26 (11.3%)	230 (100.0%)	
**Macula off**				0.0019***
No	202 (91.8%)	18 (8.2%)	220 (100.0%)	
Yes	106 (85.5%)	18 (14.5%)	124 (100.0%)	
**PVR grade**				0.1
A	213 (89.5%)	25 (10.5%)	238 (100.0%)	
B	54 (90.0%)	6 (10.0%)	60 (100.0%)	
C	41 (89.1%)	5 (10.9%)	46 (100.0%)	
**Quadrants**				0.32
N-Miss	20	3	23	
1q.	27 (93.1%)	2 (6.9%)	29 (100.0%)	
>1q.	261 (89.4%)	31 (10.6%)	292 (100.0%)	
**AXL, mm**				0.66
N-Miss	123	11	134	
Mean (SD)	24.7 (1.7)	24.9 (1.6)	24.7 (1.7)	
Range	20.1–33.2	22.7–28.2	20.1–33.2	
**Follow-Up (months)**				
N-Miss	0	1	1	
Mean (SD)	10.9 (13.1)	21.2 (14.3)	11.9 (13.5)	
Median	5.3	14.7	6.2	
Q1, Q3	1.6, 15.8	11.8, 33.6	2.0, 17.2	

Table 1 displays the demographics and clinical characteristics of patients with retinal re-detachment.  *p < 0.1, **p < 0.05, ***p < 0.01.

In Group 1, the mean age was 62.9 ± 12.9, the proportion of male patients was 202/308 (65.58%), the mean length of axial (mm) was 24.7 ± 1.7, the mean follow-up (months) was 10.9 ± 13.1 (Median: 5.3; Q1, Q3:1.6, 15.8). In Group 2, the mean age was 60.2 ± 11.1, the proportion of male patients was 18/36 (50%), the mean length of axial (mm) was 24.9 ± 1.6, the mean follow-up (months) was 21.2 ± 14.3 (Median: 14.7; Q1, Q3:11.8, 33.6). Median follow-up of total patients was 6.2 months (Q1, Q3:2.0, 17.2). There was no statistically significant difference in those variables between the two groups. There was also no statistically significant difference in lens status, PVR grade, and RD quadrants as well.

### Preoperative parameters

In preoperative parameters, 33/308 patients (10.71%) had diabetes in Group 1, while 8/36 patients (25%) in Group 2. Moreover, the macula was detached in 106/308 eyes (34.41%) in Group 1, while in Group 2, the macula was detached in 18/36 eyes (50%). Non-parametric Kaplan-Maier showed the risk factors of retinal re-detachment might involve those two variables [diabetes (p = 0.025) and macular-off (p = 0.0019)]. The results of the hazard ratios Cox regression further confirmed that diabetes and macular off before the primary surgery were associated with higher rate of retinal re-detachment outcome (diabetes: OR = 2.301, 95% CI [1.130–4.687], p < 0.05; macular-off: OR = 2.243, 95% CI [1.212–4.149], p < 0.05).

### Intraoperative parameters

In intraoperative parameters, there was no statistically significant difference in combined operations and endotamponade. In Group 1, 360° ILR was performed in 186/308 patients (60.39%), while in Group 2, 360° ILR was performed in 17/36 patients (47.22%). 17/203 eyes (8.4%) in the 360° ILR group had retinal re-detachment after primary PPV, which was significantly lower compared to the focal laser retinopexy group (19/141 eyes, 13.5%) (p < 0.05). Non-parametric Kaplan-Maier showed the risk factors of retinal re-detachment might also involve focal laser without 360° ILR (p = 0.0021). The result of the hazard ratios Cox regression further confirmed that 360° ILR was associated with a significant reduction in the odds of retinal re-detachment (OR = 0.456, 95%-CI [0.245–0.848], p < 0.05).

### Survival probabilities of retinal re-detachment

**Fig. 1:**
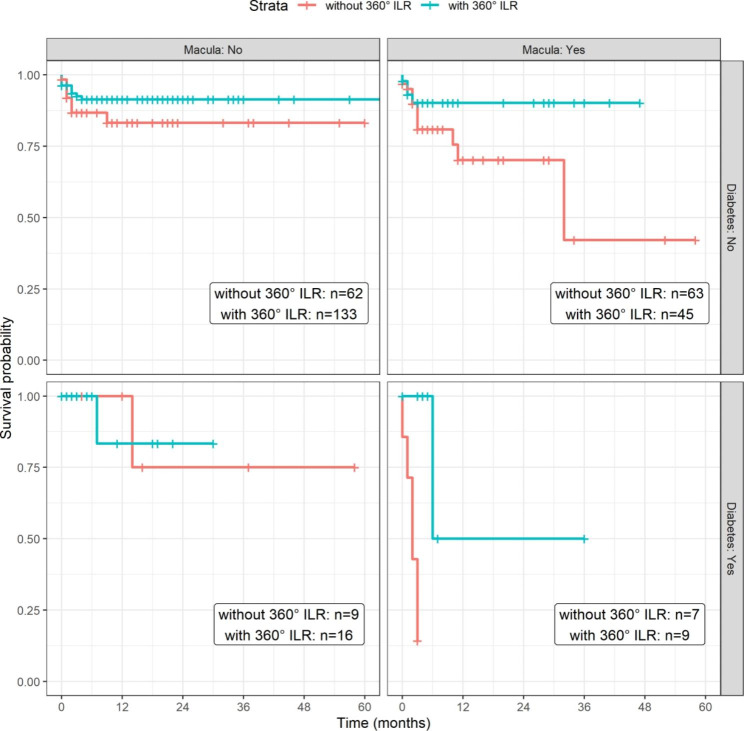
Survival probabilities of retinal re-detachment

From the previous result, we estimated observed survival probabilities for any combination of the risk factors of retinal re-detachment (shown in Fig. 1). The cumulative incidence of re-detachment at 6,12,24 and 60 months was shown in Table 2.

**Table 2:** Cumulative incidence of re-detachment at 6,12,24 and 60 months. Kaplan-Meier estimates and their 95% CI.

Month	with 360° ILR	without 360° ILR
6	9.74 [4.93;14.31]	19.54 [11.25;27.05]
12	10.78 [5.58;15.69]	25.21 [14.97;34.23]
24	10.78 [5.58;15.69]	27.55 [16.44;37.18]
60	10.78 [5.58;15.69]	37.21 [19.82;50.83]

Table 2: Cumulative incidence of re-detachment at 6,12,24 and 60 months.The differences between these cumulative incidence curves are significant (Log Rank Test p = 0.0021). The multivariate Cox regression was applied to estimate conditional hazard rates. The result of the estimation is summarized in the following table (shown in Table 2).

**Table 3:** Multivariate Cox regression

	Hazard ratio [95%-CI]
Cerclage	0.456^**^
	(0.245, 0.848)
Diabetes	2.301^**^
	(1.130, 4.687)
Macula	2.243^**^
	(1.212, 4.149)
Observations:	344
Events:	43
Wald Test:	19.22 (df = 3), p = 0
*Note*:	^*^p^**^p^***^p < 0.01

Table 3 displays the multivariate Cox regression, which estimated the conditional hazard rates.

From the regression model we calculated the predicted survival probabilities by 12 months shown in Table 4. The riskier the combination, the larger and more red was the font (shown in Table 4).

**Table 4:** Survival probabilities of retinal re-detachment

Survival probabilities of retinal re-detachment			
		**Laser Cerclage**	
**Diabetes**	**Macula**	**No**	**Yes**
No	No	**0.85**	**0.93**
No	Yes	**0.69**	**0.85**
Yes	No	**0.69**	**0.84**
Yes	Yes	**0.43**	**0.68**

Table 3 displays the survival probabilities of retinal re-detachment.

### Incidence of epiretinal membrane (ERM) formation

In regard to ERM formation, there were 18/236 cases of ERM formation in the cohort at latest follow-up. 6/118 cases occurred in the 360° ILR group (5.1%) compared to 12/118 (10.2%) in the focal laser group. 360° ILR was associated with a non-significant decrease in the odds of ERM formation (p = 0.219).

## Discussion

Retinal re-detachment after primary PPV treatment is a major complication in the management of RRD. Peripheral retinal breaks following vitrectomy, which are typically at the posterior margin of the vitreous base, are thought to be caused by traction on the vitreous base [15]. These breaks might result in retinal re-detachment after primary PPV for RRD.

Therefore, additional applications have been performed in order to reduce the risks of those peripheral retinal breaks, like intraoperative combination with scleral buckling or with 360° prophylactic intraoperative laser retinopexy. Studies showed that PPV combined with scleral buckling didn’t improve anatomic success rates. Moreover, it might have associated complications, e.g. myopia aggravation, diplopia, and formation of PVR [16–18]. 360° ILR is less invasive and can be easily performed during vitrectomy. It serves as a new shield for peripheral retinal breaks and to confine peripheral retinal re-detachment from progressing posterior. Some studies exist showing that additional application of 360° ILR during PPV might reduce the retinal re-detachment rate. Koh et al. reported a three-fold reduction (from 13.3 to 3.5%) of retinal re-detachment rate in cases of epiretinal membrane peeling and macular hole surgery [12]. Avitabile et al. reported that prophylactic 360° ILR reduced the incidence of retinal re-detachment by 58% in RRD cases after silicone oil removal [13]. Barrada et al. reported a reduction retinal re-detachment rate from 32.5 to 24% with the use of prophylactic 360° ILR [19].

In our study, 17 out of 203 eyes (8.4%) had retinal re-detachment in the 360° ILR group and 19 out of 141 eyes (13.5%) in the group treated with focal ILR. As estimated with survival analysis, the 360º ILR group had the incidence of 9.74% and focal laser 19.54% at 6 months postoperatively. At 12 months postoperatively the difference was 10.78% vs. 25.21%. The difference in survival rates was significant (p = 0.0021).These results resemble the results of Barrada`s study. Futhermore, our statistical result of survival probabilities showed that higher risks of retinal re-detachment were associated with the combination of diabetes, macular off status, and without 360° ILR treatment during the primary PPV.

There are many factors resulting in retinal re-detachment after PPV, and inflammation and responsive cells causing PVR are two main factors among them [20]. The aim of PPV in RRD was to remove such cells and also their substrates of attachment without causing an increased inflammatory response. However, the use of vitrectomy combined with laser photocoagulation has been identified as a risk factor for PVR development [21]. 360° ILR might cause the breakdown of the blood–retinal barrier with leakage of serum proteins and RPE into the intraocular fluids [22]. Therefore, this could be a source of cellular migration and proliferation resulting in epiretinal membrane formation and PVR.

In our study, patients with diabetes tended to have higher risk of retinal re-detachment, and multivariable logistic regression analysis showed that diabetes was independently associated with the occurrence of retinal re-detachment. Among all patients with diabetes, just one had signs of non-proliferative diabetic retinopathy and this patient had no retinal re-detachment. Fokkens et al. reported that advanced glycation endproducts (AGEs), such as pentosidine and 3-deoxyglucosone, which had been suggested to contribute to persistent central vitreo-retinal adhesions and lead to vitreoretinal traction, were significantly elevated in RRD patients with T2DM compared to non-diabetic RRD patients [23]. Evidence exists for a role of AGEs in the development of proliferative vitreo-retinopathy (PVR) [24]. Both AGEs and AGE-receptors were increased in the vitreous fluid of patients with PVR [25]. Moreover, AGEs could induce the expression of several cytokines that have been shown to be elevated in PVR [26–28].

Another factor for higher risk of retinal re-detachment in our study is primary RRD involving the macula. Multivariable logistic regression analysis also showed that macular detachment was independently associated with the occurrence of retinal re-detachment. These findings are in line with those Thelen et al. and Guber J et al. reported [29]–[30]. This might be partly due to the longer duration and greater extent of retinal detachment [31]. However, in our study, there was also no statistically significant difference in PVR grade and RD quadrants between the two groups. Further prospective randomized studies are required to figure out the associations between macular status and retinal redetachement.

Limitations of our study are its retrospective design and use of neither random allocation nor systematic criteria to select the patients. Therefore, selection bias cannot be ruled out. 360° ILR was used more towards the end of data collection, since at this time clinical experience and scientific reports emerged that 360° ILR might be safer concerning the retinal re-detachment rate. Although the use of multiple variable analysis in determining the primary outcome measures of this study might reduce the effect of any selection bias that may have been present, a randomized trial with systematic follow-up is still required to minimize the effects of bias.

## Conclusion

Our study demonstrates that prophylactic 360° ILR in patients treated for RRD with primary PPV leads to lower incidence of post-operative retinal re-detachment. Additionally, we found that diabetes mellitus and RRD involving the macula before the primary surgery might also be potential risk factors for a higher rate of retinal re-detachment. Given that primary single-surgery anatomical success is very important in RRD repair, we recommend the use of prophylactic 360° ILR in patients with RRD treated with PPV. Our study provides insight into the question of whether prophylactic 360° ILR should be performed routinely.

## Data Availability

The datasets used and/or analyzed during the current study are available from the corresponding author on reasonable request.

## List of abbreviations

AGE: Advanced glycation endproduct
BCVA: Best corrected visual acuity
ERM: Epiretinal membrane
ILR: Intra-operative laser retinopexy
PPV: Pars plans vitrectomy
PVR: Proliferative vitreoretinopathy
RRD: Rhegmatogenous retinal detachment
SB: Scleral buckling
